# Placental Polycyclic Aromatic Hydrocarbon (PAH) Levels Are Associated with Spontaneous Preterm Birth

**DOI:** 10.3390/ijms26178179

**Published:** 2025-08-23

**Authors:** Gwendolynn Hummel, Sohini Banerjee, Vasanta Putluri, Inaara Malick, Grace Johnson, Abu Hena Mostafa Kamal, Chandra Shekar R. Ambati, Nagireddy Putluri, Lori Showalter, Cynthia D. Shope, Joseph Hagan, Kjersti M. Aagaard, Bhagavatula Moorthy, Melissa A. Suter

**Affiliations:** 1 Department of Obstetrics and Gynecology, Baylor College of Medicine, Houston, TX 77030, USA; gwendolynn.hummel@bcm.edu (G.H.);; 2 Advanced Technology Cores, Baylor College of Medicine, Houston, TX 77030, USAabuhenamostafa.kamal@bcm.edu (A.H.M.K.); chandra.ambati@bcm.edu (C.S.R.A.);; 3 Department of Molecular and Cellular Biology, Baylor College of Medicine, Houston, TX 77030, USA; 4 Department of Pediatrics, Division of Neonatology, Baylor College of Medicine, Houston, TX 77030, USA; 5 Department of Molecular and Human Genetics, Baylor College of Medicine, Houston, TX 77030, USA; 6 HCA Healthcare and HCA Research Institute, Nashville, TN 37203, USA; 7 Maternal Fetal Care Center, Division of Fetal Medicine & Surgery, Boston Children’s Hospital, Harvard Medical School, Boston, MA 02115, USA

**Keywords:** Superfund, environmental health, pregnancy

## Abstract

While the cause of preterm birth (PTB) (i.e., delivery before 37 weeks of gestation) is likely multifactorial, ambient exposure to environmental chemicals has been postulated to play a role in its etiology. Our prior studies of exposure to polycyclic aromatic hydrocarbons (PAHs) in pregnancy have shown an increased level of placental PAH-induced bulky DNA adducts with increasing levels of PAH exposures. In this investigation, we hypothesized that higher levels of placental PAHs would be associated with an increased risk of PTB. Using gas chromatography and mass spectrometry (GC-MS/MS), we measured levels of benzo(a)pyrene (BaP), benzo(b)fluoranthene (BbF) and dibenz(a,h)anthracene (DBA) from n = 323 subjects. We found higher levels of BbF in placentae collected from preterm compared with term deliveries (mean 100.3 vs. 84.14 ng/mL, *p* = 0.038). Placental BbF levels negatively correlated with gestational age at delivery (*r_s_* = −0.171, *p* = 0.002) and placental DBA levels were higher in placentae from spontaneous PTBs compared to those that were medically indicated (mean 743.7 vs. 599.9 ng/mL, *p* = 0.049), suggesting a potentially causal role in spontaneous preterm birth. Lastly, we analyzed placental levels of each PAH in male (n = 164) and female (n = 159) gestations and found that levels of BaP are significantly higher in males (mean 204.4 vs. 169.9 ng/mL, *p* = 0.049). These studies show a potential causal role of PAH exposure in the etiology of spontaneous preterm birth.

## 1. Introduction

Preterm birth (PTB), defined as delivery at less than 37 weeks gestation, is the leading cause of infant morbidity and mortality worldwide [1,2], claiming the lives of ~1 million children under of age of 5 years in the United States and Europe annually [3]. Despite PTB being listed as a research priority by the World Health Organization and as a United Nations Sustainable Development Goal [4], the PTB rate of the United States remains the highest among industrialized nations [5,6]. Although PTB furthers health disparities [7] and carries a significant economic burden in the United States which is estimated at $25.2 billion [8], over two thirds of PTB still occur spontaneously. Therefore, a pressing need exists to explore the potential causes of spontaneous PTB.

PTB can be categorized as either spontaneous (sPTB) or medically indicated (iPTB). sPTB is labor that begins unexpectedly before 37 weeks of gestation, while iPTB is delivery of the neonate before 37 weeks gestation due to maternal or fetal complications that necessitate early delivery for the well-being of either mother, neonate or both. Such complications include placentation abnormalities (e.g., placenta previa or accreta or placental abruption), severe preeclampsia or worsening maternal hypertension, fetal growth restriction with impaired placental flow, worsening maternal diabetes, maternal morbid cardiovascular disease, and other severe maternal comorbidities. While both types of preterm delivery can lead to adverse neonatal outcomes, sPTB is poorly predicted and often recurrent. Risk factors for sPTB include a previous preterm delivery [9] and shortened cervical length [10]. The influence of fetal sex has also been implicated as a risk factor, with males being at a slightly higher risk than females for sPTB [11,12]. However, the majority of sPTBs occur in pregnancies without known risk factors and the causes remain poorly understood.

Along with periodontal disease [13,14], racial and socioeconomic factors [15,16], and genetic and metagenomic influences [17,18], environmental exposures have long been hypothesized as potential drivers of sPTB. In particular, PAHs, which are byproducts of organic combustion, have been linked to multiple adverse pregnancy outcomes [19,20,21] including reduced birth weight and an increased risk for small for gestational age neonates [22]. Exposure to PAHs occurs through many routes, including through drinking water, consumption of grilled or smoked meats, through first or secondhand tobacco smoke exposure as well as through ambient air [23].

Current research indicates that PAHs are able to cross the placenta and have been identified in cord blood collected at birth [24]. We have previously reported that benzo(a)pyrene (BaP), benzo(b)fluoranthene (BbF), and dibenz(a,h)anthracene (DBA), are found at elevated levels in the placentae of gravidae who reside near areas that have been classified as “Superfund sites.” These sites are areas designated by the Environmental Protection Agency as contaminated with chemicals which are considered hazardous to human health [19]. We also reported that increased levels of each of these PAHs is associated with increased harmful, mutagenic PAH-induced DNA bulky adducts in the placenta [19]. PAHs have been shown to alter placental gene expression, particularly through *TRIP13*, a DNA damage repair gene [25], and *CYP1A1*, an important xenobiotic-metabolizing enzyme of the placenta [19], which may play a further role in forming endogenous aryl hydrocarbon receptor ligands to aid in pregnancy maintenance [26]. These prior findings indicate that placental accumulation of PAHs may play a role in the etiology of preterm birth.

In this study, we tested the hypothesis that placental PAHs are associated with an increased risk of preterm birth. Using a total of 323 placental samples from term (n = 214) and preterm (n = 109) deliveries, we measured placental levels of BaP, BbF and DBA. We investigated associations with preterm delivery, as well as with maternal and neonatal anthropomorphic outcomes. We further used placentae from gestational aged matched preterm deliveries to determine if levels were higher from sPTBs than iPTBs. Because others have shown a sexual dimorphism of placental levels of environmental contaminants in male compared with female placentae [27,28,29,30], we also sought to determine if PAHs revealed differential levels by virtue of neonatal sex.

## 2. Results

### 2.1. Subject Demographics and Placental PAH Levels

**Figure 1:**
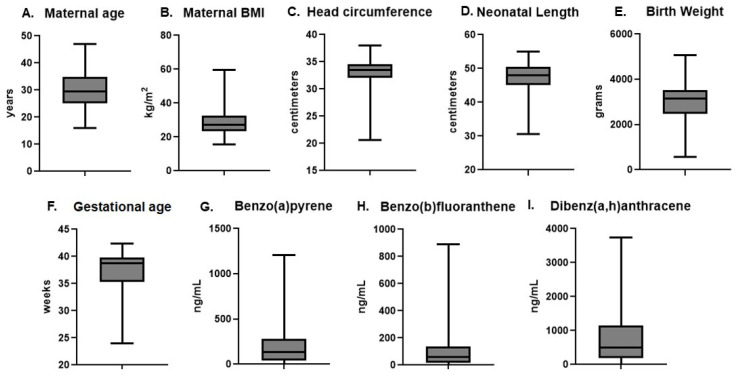
Subject characteristics and polycyclic aromatic hydrocarbon levels. Placenta samples from n = 323 subjects were included in this study. Median values for (**A**) maternal age, (**B**) maternal BMI, (**C**) head circumference, (**D**) neonatal length, (**E**) birth weight, (**F**) gestational age as well as placental levels (in ng/mL) of (**G**) benzo(a)pyrene, (**H**) benzo(b)fluoranthene and (**I**) dibenz(a,h)anthracene are represented by solid black lines. Boxes extend from the 25th to the 75th percentile of each group’s distribution of values and whiskers represent the minimum and maximum values.

A total of n = 323 placenta samples from singleton pregnancies were utilized for this study and subject characteristics are summarized in Figure 1A–F. The average ± standard deviation maternal age of our subjects was 29.9 ± 6.6 years. For subjects with pre-pregnancy weight information (n = 267), BMI was calculated; the average pre-pregnancy BMI was 28.8 ± 7.5 kg/m^2^. The average head circumference of the neonates is 32.7 ± 3.0 cm; average length is 47.4 ± 4.7 cm and the average birth weight is 2943.0 ± 877.9 g. The average gestational age at delivery for our subjects is 37.2 ± 3.8 weeks. Because this study was designed to investigate levels of PAHs associated with preterm delivery, gestational age spanned 24.0 weeks through 42.4 weeks and n = 109 placenta samples were collected from preterm deliveries as defined as delivery at less than 37 weeks gestation. A majority of our subjects self-defined as Hispanic ethnicity (n = 254, Supplemental Table S1) and both BaP and DBA were higher in subjects of Hispanic ethnicity compared with non-Hispanic ethnicity (*p* ≤ 0.03). However, given that nearly 80% of our subjects were of Hispanic ethnicity, it is unclear if this is of biological relevance and is susceptible to ascertainment bias. We did not find a difference in PAH levels across racial groups (*p* ≥ 0.356; Supplemental Table S2).

Using GC-MS/MS for measuring placental PAH levels, we determined levels of BaP, BbF and DBA from each sample. We have previously found higher levels of placental DNA adducts in association with higher levels of these PAHs [19]. DBA represented the PAH in highest abundance, with an average of 735.5 ± 693.3 ng/mL. BaP levels averaged 187.6 ± 182.9 ng/mL and BbF levels averaged 89.45 ± 94.15 ng/mL (Figure 1G–I).

### 2.2. Levels of BbF Are Significantly Associated with Preterm Delivery

**Figure 2:**
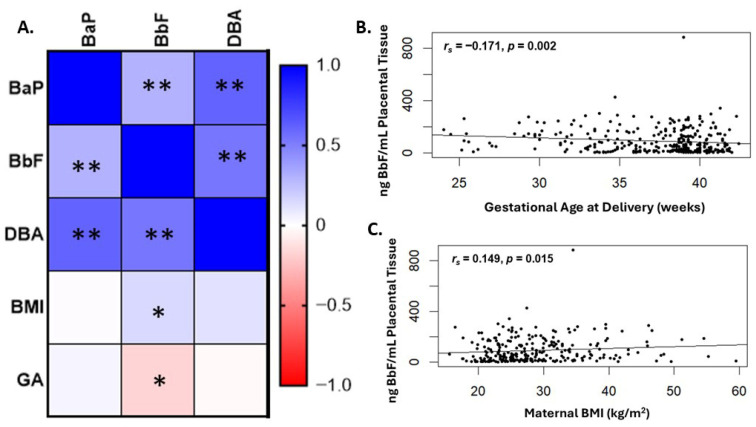
Placental levels of BbF are negatively correlated with gestational age at delivery, revealing higher levels in placentae from preterm deliveries compared with term. Using Spearman’s correlation, we investigated associations between levels of each individual PAH with continuous clinical variables. (**A**) Within the correlation matrix, significant correlations are designated with an asterisk where ** denotes *p* < 0.001 and * denotes *p* < 0.05. (**B**) BbF levels were significantly, negatively correlated with gestational age at delivery and (**C**) positively correlated with maternal BMI.

Our overall hypothesis posits that increased levels of PAHs would be associated with preterm delivery. Therefore, we investigated the association of each PAH with gestational age at delivery (Figure 2A). While BaP and DBA were not significantly associated with gestational age at delivery, levels of BbF negatively correlated with gestational age (Figure 2B, *r_s_* = −0.171, *p* = 0.002). A negative correlation indicates that higher levels of BbF early in gestation correlates with a lower gestational age. We further observed a significant positive correlation with BbF and maternal BMI (Figure 2C, *r_s_* = 0.149, *p* = 0.015). Levels of each individual PAH correlated significantly with each other (Figure 2A and Supplemental Table S3).

**Figure 3:**
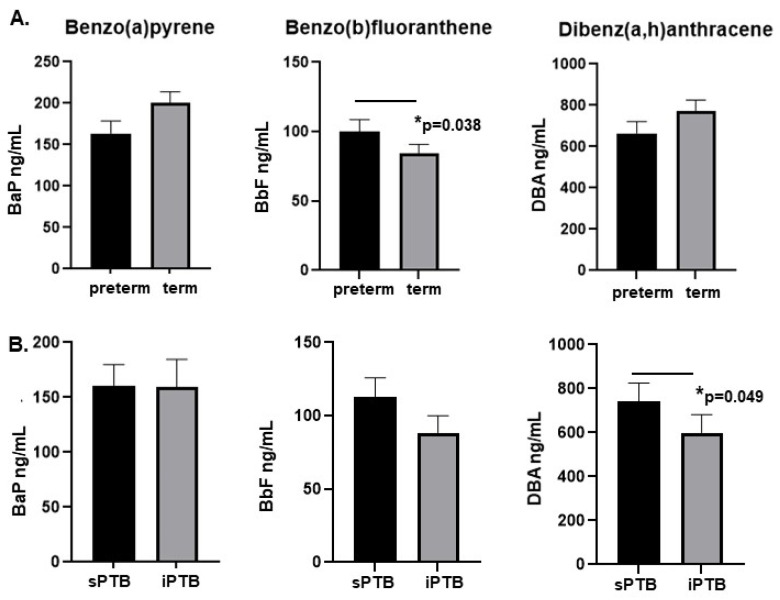
Higher placental polycyclic aromatic hydrocarbon (PAH) levels are associated with preterm delivery and spontaneous preterm birth. (**A**) Of the three PAHs tested, benzo(b)fluoranthene (BbF) levels were higher (*p* = 0.038) in placenta from preterm deliveries compared with those at term. (**B**) Comparing spontaneous (sPTB) vs. indicated preterm births (iPTB), only levels of DBA were significantly higher in sPTB (*p* = 0.049).

We further compared PAH levels with preterm delivery, stratifying into groups where term includes a gestational age of 37 weeks (n = 214) or greater and preterm is defined as less than 37 weeks gestation (n = 109). We again found levels of BbF to be significantly higher in placentae from preterm deliveries (mean 100.3 vs. 84.14 ng/mL, *p* = 0.038, Figure 3A). Neither BaP nor DBA showed a significant difference comparing term and preterm birth.

### 2.3. Levels of DBA Are Higher in Placentae from Spontaneous Preterm Deliveries Compared with Medically Indicated Preterm Delivery

Preterm delivery either can occur spontaneously or be medically indicated, due to a complication which necessitates early delivery of the fetus. We assessed whether levels of each PAH differed by virtue of type of preterm delivery. While levels of neither BaP nor BbF were significantly different, levels of DBA were significantly higher in placentae from sPTB compared with iPTB (mean 743.7 vs. 599.9 ng/mL, *p* = 0.049, Figure 3B).

### 2.4. Levels of BaP Are Higher in Placentae from Male Compared with Female Neonates

**Figure 4:**
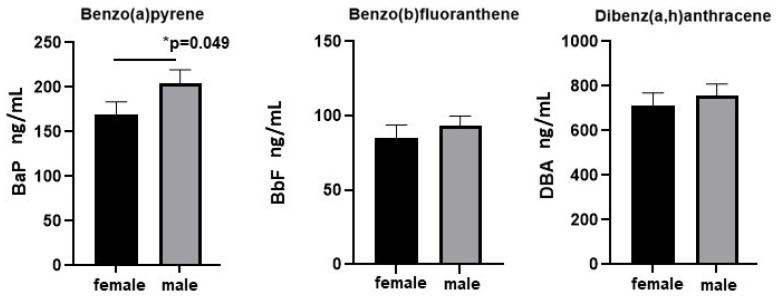
Higher placental polycyclic aromatic hydrocarbon (PAH) levels are found in placentae from males compared with females. Comparing placental PAH levels in association with neonatal sex (male vs. female), levels of BaP are significantly higher in placentae from males (*p* = 0.049).

Because sexually dimorphic differences in environmental exposures have been observed for other contaminants of concern, we sought to determine if there were differences in levels of each PAH in placentae from males and females. Our results reveal higher levels of BaP in placentae from males compared with females (mean 204.4 vs. 169.9 ng/mL, *p* = 0.049; Figure 4) while neither BbF nor DBA showed significant differences.

## 3. Discussion

PTB is associated with a remarkably high level of morbidity and mortality worldwide, and identifying modifiable risk factors that could potentially reduce the risk of preterm delivery is an urgent public and environmental health issue. In a previous study, we measured levels of harmful, mutagenic PAH-induced DNA adducts in the placenta [19] We found increased levels of these adducts with increasing levels of PAHs. Furthermore, we observed elevated placental PAH levels from preterm deliveries in women living in proximity to Superfund sites, compared to term deliveries [19]. Our hypothesis, based on our prior findings, was that increased PAH exposure during pregnancy correlates with heightened risk of PTB. The findings in this study reveal a significant and independent association between PAHs and spontaneous preterm delivery. Strengths of this study include a large sample set, inclusive of placenta samples from over 300 deliveries, spanning a wide gestational age range between 24 through 42 weeks. Within our sample set, we included placentae from both spontaneous and medically indicated preterm births. This allowed for nuanced comparisons across preterm birth subtypes. Moreover, we focused on three high-priority PAHs, selected because of their known toxicity and ubiquity, making our findings especially relevant for environmental health [31]. Limitations include insufficient power to explore racial and ethnic disparities, due to a limited number of non-Hispanic participants, which constrains our ability to assess vulnerability across populations. However, this is also a strength as it represents a cohort at risk for social and environmental disparities, which are known risk factors for spontaneous preterm birth.

Our data revealed a striking and consistent relationship between PAH exposure and PTB. A strength of this study is the direct assessment of placental levels of PAHs. A majority of the studies of PAH exposures in pregnancy utilize maternal urine levels of hydroxylated (i.e., metabolized) PAHs. In this study, we measured placental levels, which is a fetal tissue and yields insight into fetal exposures compared with maternal. Placental levels of BbF inversely and significantly correlate with gestational age at delivery. Stratifying data into term and preterm categories further supports this association with levels of BbF being higher in placentae from preterm deliveries. We have previously quantified PAHs in placentae in a smaller cohort [19], and quantities were comparable among a similar population. Because BbF is a characterized carcinogen in humans, there is no safe level of exposure in pregnancy.

Despite this significant finding, it is essential to note the low *r_s_* values of this association. The modest correlations (i.e., *r_s_* values) do not negate the importance of our significant findings. Rather, they indicate that more data is needed for more precise predictive modeling. It is our goal to expand these studies to include information on a total of 16 PAHs which have been deemed priority pollutants by the EPA [32].

For example, among samples collected from spontaneous preterm deliveries, sPTBs exhibited significantly higher levels of DBA compared to iPTB. Although DBA was not negatively correlated with overall gestational age, this finding reinforces our hypothesis that PAHs are likely significant and independent contributors to spontaneous preterm birth.

Importantly, these findings highlight the need to classify PTBs when assessing environmental risk factors. Although by definition all preterm deliveries occur before 37 weeks of gestation, their underlying causes vary. Indicated preterm delivery results from clinical necessity to prevent maternal or neonatal harm and reflects other underlying comorbidities and pathogenic diseases. Although both carcinogens, DBA forms DNA adducts at a slower pace than a similar dose of BbF [33], and BbF is more fat-soluble [34], which may play a role as placental lipid levels increase throughout gestation and are at higher levels in obese pregnancies [35]. Our observation that DBA levels are higher in sPTBs suggests a potentially different mechanistic role for individual PAHs in triggering premature labor, a finding that is clinically important and warrants deeper investigation and further study.

Interestingly, we also found that male placentae contained significantly higher levels of BaP. This aligns with previous studies indicating that male fetuses are at greater risk for preterm delivery and subsequent adverse neonatal outcomes [36] especially bronchopulmonary dysplasia (BPD) [37,38,39] and may exhibit increased placental accumulation of environmental toxins [27,28,29,30]. This lends credence to the idea that BaP and other PAHs are risk factors for PTB in pregnant women and BPD in infants. While our study was not designed to examine sex-based outcomes in depth, these sex-specific differences suggest a potential biological vulnerability that should be explored through molecular and mechanistic studies. These sex differences may be due to structural dissimilarities in the placenta, as male placentae display decreased vascular resistance compared to female placentae. Gene expression is also sexually dimorphic during distress. When perturbed, the male placenta tends to consume more energy to favor growth of both the placenta and fetus, which sacrifices plasticity and adaptability [40]. This is in contrast to the female placenta, which reacts to perturbations by adapting growth and metabolism to promote fetal survival [40]. These strategies may explain why male placentae are prone to accumulating more chemicals than female placentae [27]. Future studies are necessary to fully delve into the mechanisms behind the placental dimorphisms observed, specifically with regard to xenobiotic metabolism and regulation of the CYP P450 genes. We have previously shown that in utero exposure to maternal tobacco smoke, which contains PAHs as well as numerous other xenobiotics, decreases promoter DNA methylation and increases gene expression of the CYP1A1 gene [41]. Investigation of a difference in DNA methylation and expression between male and female placentae is warranted.

Other toxins, including organochlorine pesticides and polychlorinated biphenyls, have been reported at greater concentrations in the male fetal heart, brain, and lungs [27]. Furthermore, perfluoroalkyl substances (PFAS) have been shown to influence placental development in a sex specific manner through decreased levels of syncytiotrophoblasts in males [42]. In this way, our findings add to a growing body of literature indicating early environmental exposures may contribute to the disproportionate number of male neonates born preterm.

## 4. Materials and Methods

### 4.1. Subjects and Tissue Samples

Data and sample collection occurred under the approved protocols H-26364 and H-34056 through Baylor College of Medicine. Placental tissues were obtained by trained clinical staff from subjects enrolled in one of two studies at Baylor College of Medicine. A subset of samples (n = 276) were obtained from PeriBank, our universal perinatal database and biobank [43]. For PeriBank, subjects are enrolled at the time of delivery, and placenta samples are uniformly collected within one hour of delivery. A second subset of samples (n = 47) were obtained from subjects who participated in our longitudinal clinical trial on preterm birth (NCT0292650, K. Aagaard, PI). Placental samples were collected and aliquoted at the time of delivery and stored at −80 °C until use. For the samples in this study, exclusion criteria included fetal abnormalities, in utero fetal demise, and multiple gestations.

Clinical metadata was abstracted from the medical records by clinical research staff for the following covariates: self-identified maternal race and ethnicity, maternal age, gestational age at delivery, neonatal length, birth weight, head circumference, and preterm birth indication, if present. Maternal body mass index (BMI) was calculated using pre-pregnancy or first trimester weight when available. Preterm delivery was classified as less than 37 weeks gestation.

### 4.2. PAH Analysis of Placenta Tissue 

Measurement of PAHs in the placenta was performed as we have previously described [19]. We specifically targeted detection of the following three PAHs: benzo(a)pyrene (BaP), benzo(b)fluoranthene (BbF) and dibenz(a,h)anthracene (DBA). One hundred milligrams of tissue was utilized for extraction. Samples were homogenized with 1 mL of hexane, followed by spiking the internal standard according to tissue weight. Next, 1 mL of chloroform was added to the samples, and the samples were vortexed. The organic layer was then dried at a low boiling point using a speed vac before being reconstituted with 50 µL chloroform. The Agilent gas chromatography mass spectrometry (GC-MS/MS) system was used for the quantification of PAHs using a single electron monitoring strategy with electron impact ionization. Placental extracts (1 µL) were injected into the system for quantitation, alongside standard compounds for each PAH (BaP, BbF, DBA), ammonium acetate, and ammonium hydroxide as external standards (Sigma-Aldrich, St Louis, MO, USA). Data was analyzed with Agilent mass hunter quantitative software version 10.1, with PAH levels estimated using a standard curve. Data is reported in nanogram of PAH per milliliter of tissue (ng/mL).

### 4.3. Statistical Analyses

The Shapiro–Wilk test was used to determine normality; the distributions of BaP (*p* < 0.001), BbF (*p* < 0.001), and DBA (*p* < 0.001) all departed significantly from normality, requiring nonparametric methods to investigate associations with clinical outcomes. The Mann–Whitney test was used to examine associations between categorical variables and PAH levels while Spearman’s correlation (*r_s_*) was used for quantitative variables. Independent samples t-test was used to compare PAH levels between term and PTB, as well as sPTB and iPTB subjects.

## 5. Conclusions

Our findings identify BbF and DBA as key PAHs associated with preterm birth and early gestational age at delivery. Furthermore, BaP is specifically higher in placentae of male preterm neonates, suggesting a potential important biologic pathway distinction. Together, these results underscore the urgent need for deeper molecular investigations into PAH-induced placental changes specifically and significantly associated with spontaneous preterm birth. Moreover, we anticipate that these studies will inspire broader public health strategies aimed at minimizing maternal exposure to environmental toxins, especially in vulnerable communities such as those adjacent to PAH-containing Superfund sites. The collective impact of these studies will be to mitigate PAH exposures associated with spontaneous preterm birth.

## Data Availability

The datasets generated during and/or analyzed during the current study are available from the corresponding author upon reasonable request.

## Supplementary Materials

The following supporting information can be downloaded at: https://www.mdpi.com/article/10.3390/ijms26178179/s1.
