# Supplementary material for: Placental Polycyclic Aromatic Hydrocarbon (PAH) Levels Are Associated with Spontaneous Preterm Birth
Source: Int J Mol Sci. 2025 Aug 23;26(17):8179. doi: 10.3390/ijms26178179 (PMC12428254; doi:10.3390/ijms26178179)
Supplement: Supplementary file 1 [file ijms-26-08179-s001.zip › ijms-3755295-supplementary.pdf]

Supplemental Table S1. Hispanic ethnicity is associated with higher levels of BaP and DBA.					
**PAH (ng/mL) p-value**	**Ethnicity**	**N**	**Median**	**25th percentile**	**75th percentile**
**BaP (ng/mL) *p=0.03**	Hispanic	252	144.8	47.23	302.7
	Non-Hispanic	66	95.01	33.76	218.0
**BbF (ng/mL) p= 0.313**	Hispanic	252	61.61	17.65	138.1
	Non-Hispanic	68	48.83	13.47	138.0
**DBA (ng/mL) *p=0.002**	Hispanic	254	571.9	220.9	1223
	Non-Hispanic	68	336.6	110.6	802.7

Supplemental Table S2. PAH levels are not significantly associated with race					
	**Race**	**N**	**Median**	**25th percentile**	**75th percentile**
**BaP (ng/mL) p=0.356**	Asian	8	83.40	25.83	190.9
	Hawaiian	1	102.6	102.6	102.6
	African American	34	112.9	45.81	218.4
	White	272	142.0	45.39	290.6
	Unknown	3	31.47	26.41	109.4
**BbF (ng/mL) p=0.744**	Asian	8	46.23	23.02	97.70
	Hawaiian	1	11.24	11.24	11.24
	African American	36	66.10	16.62	135.8
	White	272	58.64	16.22	143.0
	Unknown	3	76.3	53.16	129.0
**DBA (ng/mL) p=0.701**	Asian	8	423.2	139.5	777.3
	Hawaiian	1	468.2	468.2	468.2
	African American	36	350.6	182.5	975.1
	White	274	546.8	189.8	1179
	Unknown	3	422.7	147.5	1507

**Supplemental Table S3. Significant correlations between PAHs, maternal BMI and gestational age at delivery**			
	**BaP**	**BbF**	**DBA**
**BaP**	1.00		
**BbF**	**0.300, *p<0.001**	1.00	
**DBA**	**0.610, *p<0.001**	**0.536, *p<0.001**	1.00
**BMI**	0.012, p=0.853	**0.149, *p=0.015**	0.113, p=0.065
**GA**	0.039, p=0.489	**-0.171, *p=0.002**	-0.022, p=0.699
